# Comparison of General Population, Patient, and Carer Utility Values for Dementia Health States

**DOI:** 10.1177/0272989X14557178

**Published:** 2015-01

**Authors:** Donna Rowen, Brendan Mulhern, Sube Banerjee, Rhian Tait, Caroline Watchurst, Sarah C. Smith, Tracey A. Young, Martin Knapp, John E. Brazier

**Affiliations:** Health Economics and Decision Science, School of Health and Related Research, University of Sheffield, Sheffield, UK (DR, BM, TAY, JEB); Institute of Psychiatry, King’s College London, London, UK (SB, RT, CW, MK); Brighton and Sussex Medical School, University of Brighton, Brighton, UK (SB); University College London Hospital Stroke Research Team, North Thames Clinical Research Network, London, UK (CW); Department of Health Services Research and Policy, London School of Hygiene & Tropical Medicine, London, UK (SCS); Personal Social Services Research Unit, London School of Economics, London, UK (MK)

**Keywords:** quality-adjusted life years, health-related quality of life, preference-based measures of health, dementia, Alzheimer’s disease, utilities

## Abstract

Utility values to estimate quality-adjusted life years (QALYs) for use in cost-utility analyses are usually elicited from members of the general population. Public attitudes and understanding of dementia in particular may mean that values elicited from the general population may differ from patients and carers for dementia health states. This study examines how the population impacts utility values elicited for dementia health states using interviewer-administered time tradeoff valuation of health states defined by the dementia-specific preference-based measures DEMQOL-U (patient-report) and DEMQOL-Proxy-U (carer-report). Eight DEMQOL-U states were valued by 78 members of the UK general population and 71 patients with dementia of mild severity. Eight DEMQOL-Proxy-U states were valued by 77 members of the UK general population and 71 carers of patients with dementia of mild severity. Random-effects generalized least squares regression estimated the impact of population, dementia health state, and respondent sociodemographic characteristics on elicited values, finding that values for dementia health states differed by population and that the difference varied across dementia health states. Patients with dementia and carers of patients with dementia gave systematically lower values than members of the general population that were not due to differences in the sociodemographic characteristics of the populations. Our results suggest that the population used to produce dementia health state values could impact the results of cost-utility analyses and potentially affect resource allocation decisions; yet, currently, only general population values are available for usage.

Resource allocation decisions are increasingly being informed by cost-utility analyses measuring the benefits of treatment using the quality-adjusted life year (QALY), which captures changes in both quantity and health-related quality of life (HRQL). Typically, the “quality” component of the QALY is measured using an “off-the-shelf” patient-reported generic preference-based measure valued by the general population such as the EQ-5D,^1^ HUI2,^2^ HUI3,^3^ and SF-6D.^4,5^ However, the validity and responsiveness of generic measures have been questioned for use in some conditions including dementia.^6^ Patients with dementia can exhibit impairments of recall, time perception, insight, and expressive and receptive communication. This means that patients with more severe symptoms may have particular difficulties in making self-reports of their own HRQL. For these patients, proxy reporting may be necessary. The DEMQOL system (self-reported DEMQOL and proxy [carer]–reported DEMQOL-Proxy) was specifically developed and validated in a population with dementia and can be used together to measure the HRQL of patients with mild, moderate, and severe dementia.^7,8^ DEMQOL-U and DEMQOL-Proxy-U are recently developed dementia-specific preference-based measures derived from DEMQOL and DEMQOL-Proxy. These measures were valued by members of the UK general population and can be used to estimate QALYs for use in cost-utility analyses.^9–11^

Utility values for preference-based measures can be elicited from a number of populations including members of the general population, patients affected by a particular illness, carers, and health care professionals. Typically, utility values for use in economic evaluations are elicited from the general population in accordance with recommendations by agencies such as the National Institute for Health and Care Excellence (NICE)^12^ and the Canadian Agency for Drugs and Technologies in Health.^13^ General population values are typically recommended, as health care is publicly funded (partly or fully, depending on a country’s health system). Furthermore, members of the general population, unlike patients, have no vested interest, as they do not have prior knowledge of their own future health states. In addition, the practicality of undertaking general population studies rather than separate patient valuation studies across all patient groups is an advantage. However, members of the general population may not fully understand the impact of hypothetical health states, whereas patients who have experienced similar health states may be better able to provide more accurate valuations. The source of elicited utility values is important because the general population may provide different values to other populations. This means that the choice of whose values may affect the values obtained and can impact the results of economic evaluations based on such data.^14^

A review and meta-analysis found that patients and the general population provide almost identical values for hypothetical health states.^15^ However, the results differed by health state description and elicitation technique, where patients gave significantly lower values than the general population when values were elicited using time tradeoff (TTO). The results also differed when patients valued their own health state but the general population valued a hypothetical state, where patients gave higher values. Reasons for observed differences in general population and patient values include the following: 1) different populations value different health states due to differences in their understanding or interpretation of the description; 2) different populations have different scales of measurement due to a response shift; and 3) patients may take into account adaptation to the health state, whereas members of the general population typically do not consider adaptation. In a valuation survey, all participants have to imagine what it is like to be in a hypothetical state; yet, members of the general population have less to base this on than patients who may have experienced similar health states and have a greater understanding of what it is like to experience that health state. This also means that the patient may consider additional information about the experience of the health state that is not included in the health state description. The patient may have knowledge of adaptation to the health state or similar states but as a result may be unable to imagine full health or mild health states and may have lowered their expectations as a result. In addition, members of the general population and patients may differ in their sociodemographic characteristics, and this may also affect how they value health states.

The literature examining differences between values elicited by patients and members of the general population has mainly focused on physical health states; yet, the relationship may differ for conditions affecting mental health and cognition. One work reporting on 2 studies found that people experiencing a range of different health states gave mental health a greater weight than physical health, whereas members of the general public trying to imagine the same health states gave physical health a higher weight than mental health.^16^ Another article found that patients with depression gave lower utility values for depression health states than the general population.^17^ One article found that patients with epilepsy did not give significantly different utility values for epilepsy health states than the general population, with the exception of a significantly higher value for the worst possible state.^18^ Public attitudes and the understanding of dementia in particular may mean that patient and general population values will differ. In addition, values elicited from carers of patients with dementia may differ from values elicited from the general population, as carers have experience of how the condition impacts the patient. At the same time, carers may be in a better position to value states than patients, particularly as cognitive impairment becomes more severe.

Previous studies have been limited in a number of ways. They usually fail to adjust for sociodemographic differences between populations. It is important to control for sociodemographic characteristics of the samples^19^ because elicited TTO values can vary by sociodemographic characteristics,^20^ meaning that some of the variation in values across populations may be due to differences in the sociodemographic characteristics of the population. The literature is also limited, as most studies ask respondents to value only a small number of states. This means that it cannot be determined whether there are systematic differences in values elicited from different populations across all health states and whether the difference varies by health state severity. Even studies that valued a large sample of health states have not examined whether differences vary by severity. For example, one study estimated different value sets for patients and members of the general population for the EQ-5D but, when using the data from both populations to estimate a combined value set, did not include interaction effects for the severity levels of each dimension.^21^ However, one study reported that values elicited using a visual analog scale with members of the general population with no health problems, mild health problems, and moderate health problems did vary by health state severity.^19^

To address these general limitations and to investigate the difference in values elicited for dementia health states from members of the general population, patients with dementia, and their carers, we completed a between-subject study comparing dementia health state values from samples of the general population, patients with mild dementia, and carers of patients with mild dementia for a range of dementia health states of differing severity. Dementia health states were defined using DEMQOL-U and DEMQOL-Proxy-U.^9,10^ Regression analysis was used to explore whether population, dementia health state severity, and respondent sociodemographic characteristics impact elicited utility values.

## Methods

### Dementia Health State Description

Dementia health state descriptions were generated using the DEMQOL-U and DEMQOL-Proxy-U classification systems^9,10^ (Table 1). DEMQOL-U was derived from the DEMQOL patient-reported measure of HRQL and has 5 dimensions (positive emotion, memory, relationships, negative emotion, and loneliness), each with 4 levels of severity. DEMQOL-Proxy-U was derived from the carer-reported DEMQOL-Proxy measure of HRQL and has 4 dimensions (positive emotion, memory, appearance, and negative emotion), each with 4 levels of severity. The classification systems of DEMQOL-U and DEMQOL-Proxy-U differ, as they were each derived from a different parent measure that has different domains and items (although some are common across both measures). DEMQOL and DEMQOL-Proxy have different items because in the development studies, items were found to work differently for people with dementia and family carers. The final instruments included those items that worked best for the target group completing the instrument, but both measure the HRQL of the person with dementia. Both DEMQOL and DEMQOL-Proxy were derived by the same research team at the same time using the same methodology, as were the utility measures that were derived from them. Each dementia health state is made up of each dimension at a selected level of severity; for example, DEMQOL-U health state 11111 is made up of all dimensions at the lowest level of severity, and DEMQOL-U health state 44444 is made up of all dimensions at the highest level of severity.

**Table 1:** DEMQOL-U and DEMQOL-Proxy-U Health State Classification Systems

DEMQOL-U	DEMQOL-Proxy-U
Positive emotion	Positive emotion
1. I feel cheerful a lot	1. I feel lively a lot
2. I feel cheerful quite a bit	2. I feel lively quite a bit
3. I feel cheerful a little	3. I feel lively a little
4. I do not feel cheerful at all	4. I do not feel lively at all
Memory	Memory
1. I do not worry at all about forgetting things that happened recently	1. I do not worry at all about forgetting what day it is
2. I worry a little about forgetting things that happened recently	2. I worry a little about forgetting what day it is
3. I worry quite a bit about forgetting things that happened recently	3. I worry quite a bit about forgetting what day it is
4. I worry a lot about forgetting things that happened recently	4. I worry a lot about forgetting what day it is
Relationships	Appearance
1. I do not worry at all about making myself understood	1. I do not worry at all about keeping myself looking nice
2. I worry a little about making myself understood	2. I worry a little about keeping myself looking nice
3. I worry quite a bit about making myself understood	3. I worry quite a bit about keeping myself looking nice
4. I worry a lot about making myself understood	4. I worry a lot about keeping myself looking nice
Negative emotion	Negative emotion
1. I do not feel frustrated at all	1. I do not feel frustrated at all
2. I feel frustrated a little	2. I feel frustrated a little
3. I feel frustrated quite a bit	3. I feel frustrated quite a bit
4. I feel frustrated a lot	4. I feel frustrated a lot
Loneliness	
1. I do not feel lonely at all	
2. I feel lonely a little	
3. I feel lonely quite a bit	
4. I feel lonely a lot	

### Selection of Dementia Health States

A sample of 8 dementia health states for each of the 2 measures was selected that represented the full severity range of each classification system while ensuring that there was a mixture of severity levels for each dimension across the dementia health states. For each measure, all respondents valued the same states.

### Samples

Dementia health states derived from each measure were valued by members of the general population in accordance with recommendations from NICE^12^ and also by the population that completes the questionnaire and was involved in the initial development of the parent measure (DEMQOL and DEMQOL-Proxy).^7,8^ This means that DEMQOL-U health states were also valued by people with dementia and that DEMQOL-Proxy-U health states were also valued by carers of people with dementia. The sample size of each population for each measure was chosen to ensure sufficient power for a comparison of mean dementia health state values across the different populations for each measure using simple *t* tests. This required a total of 71 completed interviews per population per classification system, assuming a power of 0.8, significance level of 0.05, standard deviation of 0.3, and expected difference of 0.1.

#### General population

The sample was a subset of 155 respondents from the 600 respondents included in the general population valuation study of DEMQOL-U and DEMQOL-Proxy-U.^9^ The sample of respondents used here consisted of those who valued the 8 DEMQOL-U and 8 DEMQOL-Proxy-U health states also valued by patients and carers. Interviewers systematically rotated combinations of dementia health states in the interviews to ensure that the subsample used here was a random sample of the 600 and hence representative of the general population. The sample of 600 respondents was obtained by sampling households in urban and rural areas in North England using the AFD Names and Numbers version 3.1.25 database (AFD Software Ltd, Ramsey, UK). The sample was then balanced to the UK population according to geodemographic profiles. Households that were sampled were first contacted by post using a letter introducing the survey and informing them that interviewers would be in their area. Trained and experienced interviewers then visited sampled households to request participation in the survey and to arrange a suitable appointment for a home interview. The interviewers visited each sampled household at different times of the week if there was no response.

#### Patients with dementia and carers

Patients with an established clinical diagnosis of dementia of a mild severity (defined as a Mini-Mental State Examination [MMSE] score >18)^22^ and their carers were recruited by 2 research workers (authors RT and CW) from the clients of clinical teams from the Mental Health of Older Adults and Dementia Service at the South London and Maudsley NHS Foundation Trust including the following: the Croydon Memory Service, the Southwark and Lambeth Memory Service, and Community Mental Health Teams in Croydon and Lewisham. Clinical diagnoses were made following a detailed multidisciplinary assessment,^23^ and diagnostic data were obtained from the clinical record. Letters introducing the survey and information sheets were sent by post to all patients referred as suitable for participation in the study by their care coordinators. Research workers then contacted the patients by telephone to arrange a suitable appointment for a home interview with the interviewers visiting as a pair. If both the patient with dementia and their carer were participating, interviews were conducted simultaneously in a different room where possible. The research workers received the same training as the interviewers who carried out the general population survey.

### Valuation Task

The TTO technique was chosen in accordance with the UK valuation of the EQ-5D using the Measurement and Valuation of Health (MVH) protocol, including a visual prop designed by the MVH group (University of York)^1^ with an upper anchor of “full health.” This determines the point at which respondents are indifferent between 10 years in the dementia health state and x years in full health. For states regarded as worse than dead, indifference is determined between death and y years in the dementia health state, followed by x number of years in full health (where x + y = 10). This meets the reference case recommended by NICE.^12^ At the start of the interview, respondents self-completed the EQ-5D; the patient sample completed the DEMQOL-U, and the carer sample completed the DEMQOL-Proxy-U to familiarize themselves with the classification system used in the valuation task. General population respondents then ranked 8 dementia health states plus “full health” and “dead” in order from best to worst. Patients with dementia and carers did not undertake the ranking task, as it requires the consideration of all 10 health states (8 dementia health states and full health and dead) simultaneously, and given that dementia is a condition that affects memory, this was considered inappropriate. The same protocol was used for both patients with dementia and carers for consistency and to ensure that patients with dementia felt that they were treated the same as their carers. The general population sample was not informed that the survey was about dementia.

All respondents undertook a practice TTO task using a hypothetical “practice” state to familiarize themselves with the process. All respondents then valued 8 DEMQOL-U or DEMQOL-Proxy-U health states using TTO, and for general population respondents, these were the dementia health states that they had previously ranked. At the end of the interview, respondents rated how difficult they found the rank (where appropriate) and TTO tasks and answered questions about sociodemographic characteristics and health service use. Interviewers reported whether they thought the respondent had understood the TTO tasks (understood and performed exercises easily/some problems but seemed to understand the exercises in the end/doubtful whether the respondent understood the exercises).

The general population survey was approved by the ScHARR Research Ethics Committee at the University of Sheffield. The patient with dementia and carer survey was approved by the London Research Ethics Committee.

### Analysis

A factorial analysis of variance, estimated using a generalized linear model, was performed to assess significant differences in respondent characteristics across the different populations. Descriptive statistics of dementia health state values across the different populations are presented by measure. Simple *t* tests were used to compare mean dementia health state values across the different populations.

### Regression Analysis

Regression analysis was used to examine the impact of population and dementia health state severity on elicited utility values while controlling for respondent sociodemographic characteristics. The standard model specification was

$$y_{ij}=\alpha +{\beta h}_{j}+{\gamma p}_{i}+{\theta q}_{\mathrm{ij}}+{\delta s}_{i}+\varepsilon_{ij},$$

where i=1,2,...,n represents individual respondents and j=1,2,...,m represents the 8 DEMQOL-U or DEMQOL-Proxy-U health states, *y* represents the TTO utility value, **h** represents the vector of dummy variables for the dementia health states, *p* represents the dummy variable capturing the population (where *p* = 1 for patients with dementia and carers), **q** represents the vector of interaction terms to capture the population and dementia health state (e.g., in the DEMQOL-U regression analysis, a dummy variable for state 12231 valued by patients equals 1 for state 12231 valued by a patient with dementia), **s** represents the vector of sociodemographic characteristics, and $\varepsilon_{ij}$ represents the error term. Random-effects and fixed-effects generalized least squares models were estimated, as all respondents have multiple observations.^24^ The reference dementia health state in the regression analysis is measure-specific full health, health state 11111 for DEMQOL-U, and health state 1111 for DEMQOL-Proxy-U.

Three model specifications were estimated: first, regressions were estimated using dementia health state dummies and population as explanatory variables; second, interaction terms capturing population and dementia health state effects were added; and third, sociodemographic characteristics were added. This procedure was undertaken to determine whether model performance was improved by including interactions that allow for the impact of population to vary by dementia health state in the model specification and to determine whether population and severity effects were important when respondent sociodemographics were controlled for (as some differences in values across populations may be due to the differences in the sociodemographic composition of the samples rather than the population per se). A further model was estimated using a sample that excluded respondents whose understanding of the TTO task was doubted by the interviewers.

The performance of regression models was assessed using within *R*^2^, between *R*^2^, overall *R*^2^, root mean squared error of predictions, and Wald χ^2^. STATA version 11 (StataCorp, College Station, TX) was used for all regression analyses, and SPSS version 15 (SPPS Inc, Chicago, IL) was used for the descriptive statistical analysis.

## Results

### Samples

Recruitment of the general population sample is summarized elsewhere,^9^ and recruitment of the patient sample is also summarized in another work.^11^ Patients with dementia and carers were recruited between 7 November 2010 and 23 May 2011, during which the clinical teams referred 196 patients diagnosed with dementia. Of these, 93 patients and 73 carers agreed to be interviewed and were assessed as suitable by the research workers. Recruitment continued until 71 patients and 71 carers had completed the interview. During the process, 21 partial interviews were undertaken, 19 with patients and 2 with carers, and 3 patients were not able to participate at the time of the arranged interview. Partial interviews were interviews terminated before the end of the TTO tasks due to respondent fatigue, misunderstanding, or distress, and these 21 interviews are therefore excluded from the analysis. Some patients were interviewed without carers, and a few carers were interviewed without patients, meaning the completed interviews contained 49 patient/carer dyads.

One general population respondent was excluded for valuing all states identically but lower than one, as this indicates that they could not distinguish between the different dementia health states and hence may have misunderstood the task. No patients with dementia or carers were excluded on this basis.

There were significant differences between the general population and patient samples valuing the DEMQOL-U measure for age, employment status, education, and interviewers reporting that it was doubtful that the respondent understood the TTO tasks (Table 2). As expected, the patient sample was significantly older, with a mean (±standard deviation) age of 78.4 ± 7.7 years in comparison to 49.6 ± 17.0 years for the general population sample. The patient sample had a much higher proportion of retired individuals and those for which secondary school was their highest level of education. Additionally, the patient sample had a higher proportion of respondents in which the interviewers reported that it was doubtful that respondents understood the TTO tasks. The EQ-5D scores of their own health were similar between both samples. Although this might appear surprising, the validity of using generic preference-based measures in dementia has been questioned, with one study finding that 48% of people with dementia self-reported being in full health using the EQ-5D.^25^ The mean MMSE score of patients was 23.5 ± 2.8, with a range from 19 to 30.

**Table 2:** Characteristics of Respondents by Measure and Population

	DEMQOL-U			DEMQOL-Proxy-U		
	General Population (*n* = 78)	Patient Population (*n* = 71)	*P* Value (ANOVA)	General Population (*n* = 77)	Carer Population (*n* = 71)	*P* Value (ANOVA)
Age, mean ± standard deviation, y	49.6 ± 17.0	78.4 ± 7.7	<0.001	50.7 ± 16.2	69.8 ± 12.7	<0.001
Age distribution,^a^ %			<0.001			<0.001
18-40 y	30.8	0.0		32.5	0.0	
41-65 y	48.7	8.5		45.4	33.8	
>65 y	20.5	91.5		22.1	66.2	
Female, %	52.6	54.9	0.77	51.9	63.4	0.16
Married/partner, %	66.7	62.0	0.55	74.0	85.9	0.07
Employment status,^a^ %			<0.001			0.002
Employed or self-employed	53.8	2.8		45.5	29.6	
Unemployed	2.6	0.0		2.6	0.0	
Long-term sick	3.8	0.0		1.3	0.0	
Full-time student	1.3	0.0		5.2	0.0	
Retired	24.4	90.1		33.8	62.0	
Own home outright or with mortgage, %	79.5	87.3	0.20	72.7	94.4	<0.001
Secondary school is highest level of education, %	34.6	50.7	0.05	35.1	38.0	0.71
Interviewer reported that it was doubtful that respondent understood TTO tasks, %	1.3	12.7	0.001	0.0	7.0	0.02
Duration of interview, mean ± standard deviation, min	28.5 ± 9.0	29.4 ± 12.0	0.583	28.0 ± 8.0	22.5 ± 7.4	<0.001
EQ-5D score, mean ± standard deviation	0.87 ± 0.19	0.85 ± 0.14		0.79 ± 0.25	0.78 ± 0.19	

Notes: Adapted from Mulhern and others.^11^ TTO = time tradeoff.

a. To adhere to statistical assumptions, categories are merged if there are 0 observations or fewer than 5 expected observations, and χ^2^ *P* values are reported.

There were significant differences between the general population and carer samples valuing the DEMQOL-Proxy-U measure for age, employment status, marital status, home ownership, and interviewers reporting that it was doubtful that the respondent understood the TTO tasks (Table 2). The carer sample was significantly older, with a mean age of 69.8 ± 12.7 years in comparison to 50.7 ± 16.2 years for the general population sample. The carer sample had a lower proportion of employed and unemployed individuals and students but a higher proportion of retired individuals, married individuals, and individuals owning their own home. The carer sample had a higher proportion of respondents in which the interviewers reported that it was doubtful that they understood the TTO tasks. The EQ-5D scores of their own health were similar between both samples.

### Descriptive Statistics of Dementia Health State Values

Descriptive statistics of observed TTO values for DEMQOL-U show that TTO values elicited from patients have significantly lower mean and lower median values for every dementia health state than the equivalent values elicited from the general population (Table 3). The patient sample also has a larger range of mean utility values (0.816 to −0.023) than the general population sample (0.955 to 0.190). The ordering of the dementia health states by utility value differs for the patient and general population samples. However, for each sample, the ordering of the dementia health states is not logically inconsistent with the severity of the dementia health state, as for many states, it is not possible to determine a logical ordering because one state is not consistently better across all dimensions.

**Table 3:** Descriptive Statistics of Observed TTO for DEMQOL-U

Sample	General Population (*n* = 78)		Patient Population (*n* = 71)		
Health State	Mean ± SD	Median	Mean ± SD	Median	Mean Difference
11111	0.955 ± 0.153	1.000	0.816 ± 0.241	0.900	0.139^a^
12231	0.894 ± 0.160	1.000	0.633 ± 0.297	0.700	0.261^a^
32143	0.673 ±0.302	0.663	0.399 ± 0.398	0.500	0.274^a^
41212	0.611 ± 0.364	0.625	0.436 ± 0.347	0.500	0.175^a^
23424	0.566 ± 0.340	0.600	0.435 ± 0.327	0.500	0.131^b^
44221	0.544 ± 0.367	0.525	0.327 ± 0.439	0.475	0.217^a^
43442	0.403 ± 0.412	0.475	0.161 ± 0.455	0.225	0.241^a^
44444	0.190 ± 0.484	0.250	−0.023 ± 0.456	0.000	0.213^a^

Notes: Adapted from Mulhern and others.^11^ SD = standard deviation; TTO = time tradeoff.

a. *t* test is significant at the 1% level.

b. *t* test is significant at the 5% level.

Descriptive statistics of observed TTO values for DEMQOL-Proxy-U show that TTO values elicited from carers also have significantly lower mean and median values for every dementia health state than the equivalent values elicited from the general population (with the exception that the difference is not significant for the best state) (Table 4). The carer sample also has a larger range of mean utility values (0.857–0.049) than the general population sample (0.918–0.370). The ordering of the dementia health states by utility value differs for the carer and general population samples, but the ordering is logically consistent for each population.

**Table 4:** Descriptive Statistics of Observed TTO for DEMQOL-Proxy-U

Sample	General Population (*n* = 77)		Carer Population (*n* = 71)		
Health State	Mean ± SD	Median	Mean ± SD	Median	Mean Difference
1111	0.918 ± 0.185	1.000	0.857 ± 0.211	1.000	0.061
1222	0.869 ± 0.174	0.925	0.731 ± 0.251	0.700	0.138^a^
3112	0.804 ± 0.255	0.925	0.666 ± 0.313	0.725	0.138^a^
1341	0.807 ± 0.236	0.900	0.640 ± 0.250	0.700	0.167^a^
3234	0.672 ± 0.272	0.700	0.458 ± 0.361	0.500	0.215^a^
2424	0.638 ± 0.341	0.700	0.464 ± 0.364	0.500	0.174^a^
4411	0.580 ± 0.395	0.625	0.380 ± 0.406	0.500	0.200^a^
4444	0.370 ± 0.482	0.400	0.049 ± 0.465	0.000	0.321^a^

Notes: Adapted from Mulhern and others.^11^ SD = standard deviation; TTO = time tradeoff.

a. *t* test is significant at the 1% level.

### Regression Analysis

Models using a range of sociodemographic variables as explanatory variables were estimated, and the best models (using significance of coefficients, within *R*^2^, between *R*^2^, overall *R*^2^, root mean squared error of predictions, and Wald χ^2^) had only 1 significant coefficient at the 5% level across all models estimated (dummy variable for female for DEMQOL-U for the model excluding respondents whose understanding of the TTO task was doubted by the interviewers), and this had a minimal impact on model performance. For this reason, models including sociodemographic characteristics are not presented here. Models without sociodemographic characteristics are presented in Tables 5 and 6 for DEMQOL-U and DEMQOL-Proxy-U, respectively. Random-effects models were reported, as the Hausman test confirmed for both measures that fixed-effects models would produce similar estimates at reduced efficiency.

**Table 5:** Regression Analysis of DEMQOL-U Health State Values across General Population and Patient Respondents

	Model 1	Model 2	Model 3
States			
12231	−0.119^a^ (–0.179 to −0.059)	−0.0606 (–0.143 to 0.022)	−0.0606 (–0.143 to 0.021)
32143	−0.346^a^ (–0.406 to −0.286)	−0.281^a^ (–0.364 to −0.198)	−0.281^a^ (–0.363 to −0.199)
41212	−0.361^a^ (–0.421 to −0.301)	−0.344^a^ (–0.426 to −0.261)	−0.344^a^ (–0.426 to −0.262)
23424	−0.385^a^ (–0.444 to −0.325)	−0.388^a^ (–0.471 to −0.305)	−0.388^a^ (–0.470 to 0.306)
44221	−0.448^a^ (–0.508 to −0.388)	−0.411^a^ (–0.493 to −0.328)	−0.411^a^ (–0.493 to −0.329)
43442	−0.601^a^ (–0.661 to −0.541)	−0.552^a^ (–0.635 to −0.469)	−0.552^a^ (–0.634 to −0.470)
44444	−0.800^a^ (–0.860 to −0.740)	−0.765^a^ (–0.847 to −0.682)	−0.765^a^ (–0.847 to −0.683)
Patient population	−0.206^a^ (–0.290 to −0.123)		
Patient interaction terms			
11111 × patient		−0.139^b^ (–0.254 to −0.023)	−0.119^b^ (–0.234 to −0.003)
12231 × patient		−0.261^a^ (–0.376 to −0.146)	−0.248^a^ (–0.363 to −0.132)
32143 × patient		−0.274^a^ (–0.389 to −0.159)	−0.249^a^ (–0.364 to 0.133)
41212 × patient		−0.175^a^ (–0.291 to −0.060)	−0.177^a^ (–0.291 to −0.061)
23424 × patient		−0.131^b^ (–0.246 to 0.016)	−0.138^b^ (–0.253 to −0.022)
44221 × patient		−0.217^a^ (–0.332 to −0.101)	−0.194^a^ (–0.310 to −0.079)
43442 × patient		−0.241^a^ (–0.357 to −0.126)	−0.224^a^ (–0.339 to −0.108)
44444 × patient		−0.213^a^ (–0.328 to −0.097)	−0.207^a^ (–0.322 to −0.091)
Constant	0.987^a^ (0.917 to 1.057)	0.954^a^ (0.875 to 1.034)	0.954^a^ (0.878 to 1.031)
Observations	1192	1192	1120
Number of individuals	149	149	140
Within *R*^2^	0.478	0.483	0.489
Between *R*^2^	0.137	0.137	0.136
Overall *R*^2^	0.341	0.385	0.358
Root mean squared error	0.251	0.251	0.242
Wald χ^2^	971.76	986.02	944.96

Notes: 95% confidence intervals are in parentheses. The reference state is 11111 valued by the general population. Model 1 includes as explanatory variables dementia health state dummies and population. Model 2 also includes interaction terms capturing population and dementia health state effects. Model 3 has the same specification as model 2 and excludes 9 respondents whose understanding of the time tradeoff task was doubted by the interviewers. The patient population variable is a dummy variable for patients.

a. Significant at 1% level.

b. Significant at 5% level.

**Table 6:** Regression Analysis of DEMQOL-Proxy-U Health State Values across General Population and Carer Respondents

	Model 4	Model 5	Model 6
States			
1222	−0.0866^a^ (–0.143 to −0.031)	−0.050 (–0.127 to 0.027)	−0.0497 (–0.126 to 0.027)
3112	−0.151^a^ (–0.207 to −0.095)	−0.114^a^ (–0.191 to −0.037)	−0.114^a^ (–0.191 to −0.037)
1341	−0.163a (–0.219 to −0.106)	−0.112^a^ (–0.189 to −0.035)	−0.112^a^ (–0.188 to −0.035)
3234	−0.320^a^ (–0.376 to −0.264)	−0.246^a^ (–0.323 to −0.169)	−0.246^a^ (–0.322 to −0.169)
2424	−0.335^a^ (–0.391 to −0.279)	−0.280^a^ (–0.357 to −0.203)	−0.280^a^ (–0.357 to −0.204)
4411	−0.405^a^ (–0.461 to −0.349)	−0.338^a^ (–0.415 to −0.261)	−0.338^a^ (–0.415 to −0.262)
4444	−0.673^a^ (–0.729 to −0.617)	−0.548^a^ (–0.625 to −0.471)	−0.548^a^ (–0.625 to −0.471)
Carer population	−0.177^a^ (–0.250 to −0.103)		
Carer interaction terms			
1111 × carer		−0.061 (–0.164 to 0.043)	−0.0557 (–0.162 to 0.051)
1222 × carer		−0.138^a^ (–0.242 to −0.034)	−0.130^b^ (–0.236 to −0.023)
3112 × carer		−0.138^a^ (–0.242 to −0.034)	−0.123^b^ (–0.229 to −0.016)
1341 × carer		−0.167^a^ (–0.271 to −0.063)	−0.150^a^ (–0.257 to −0.044)
3234 × carer		−0.215^a^ (–0.319 to −0.111)	−0.215^a^ (–0.321 to −0.108)
2424 × carer		−0.174^a^ (–0.278 to −0.070)	−0.168^a^ (–0.275 to −0.062)
4411 × carer		−0.200^a^ (–0.304 to −0.095)	−0.192^a^ (–0.299 to −0.086)
4444 × carer		−0.321^a^ (–0.425 to −0.217)	−0.325^a^ (–0.432 to −0.219)
Constant	0.974^a^ (0.911 to 1.037)	0.918^a^ (0.846 to 0.990)	0.918^a^ (0.846 to 0.990)
Observations	1184	1184	1144
Number of individuals	148	148	143
Within *R* ^2^	0.478	0.483	0.450
Between *R* ^2^	0.137	0.137	0.120
Overall *R* ^2^	0.341	0.385	0.321
Root mean squared error	0.236	0.237	0.236
Wald χ^2^	971.76	986.02	827.53

Notes: 95% confidence intervals are in parentheses. The reference state is 1111 valued by the general population. Model 4 includes as explanatory variables dementia health state dummies and population. Model 5 also includes interaction terms capturing population and dementia health state effects. Model 6 has the same specification as model 5 and excludes 5 respondents whose understanding of the time tradeoff task was doubted by the interviewers. The carer population variable is a dummy variable for carers.

a. Significant at 1% level.

b. Significant at 5% level.

Dementia health state dummy variables were significant at the 1% level across all models, with the exception of state 12231 in models 2 and 3 and state 1222 in models 5 and 6. The dummy variable representing population in models 1 and 4 was significant at the 1% level and demonstrated that patients and carers value states significantly lower than the general population.

The inclusion of interaction terms in models 2 and 5 reflecting the interaction between the specific dementia health state and the patient or carer population improved model performance as measured using within and overall *R* ^2^ and Wald χ^2^ and reduced the absolute size of the coefficient for the state dummy variables, with the exception of DEMQOL-U state 23424 in model 2. The interaction effects had negative coefficients, meaning that patients and carers had an additional utility decrement for each state above the utility decrement reflected by the coefficients of the dementia health state dummy variables. The size of the interaction coefficients varied by dementia health state, meaning that the impact of the population differs by state severity and composition. Interaction effects reflecting the interaction between patient valuation and dementia health state for DEMQOL-U had larger coefficients for all dementia health states with more severe problems in the (fourth) negative emotion dimension (states 12231, 32143, 43442, and 44444). Interaction effects reflecting the interaction between carer valuation and dementia health state for DEMQOL-Proxy-U had larger coefficients for all dementia health states with more severe problems in 1 or more dimensions (states 1341, 2424, 3234, 4411, and 4444), with a noticeably larger coefficient for the worst state (state 4444). The exclusion of respondents whose understanding of the TTO task was doubted by the interviewers in models 3 and 6 had no noticeable impact on coefficients for dementia health state severity and interaction effects.

## Discussion

The TTO utility values for dementia health states differed depending upon whether the values were elicited from patients with dementia and the carers of patients with dementia or members of the general population. It is striking that patients and carers gave systematically lower utility values for every dementia health state than members of the general population. This is contrary to the overall findings of a recent review and meta-analysis that found that patient and general population preferences for hypothetical health states were almost identical but is consistent with the sensitivity analysis that found that patient values were significantly lower than general population values when using the TTO elicitation technique to value hypothetical health states.^15^

Descriptive statistics of observed values and regression analysis indicated that utility values across populations differed for different dementia health states and indicated a difference in the ordering of the dementia health states by utility value across populations. The differences between patient/carer and general population values for measure-specific full health are surprising, given that there are no health problems in measure-specific full health. The difference in the ordering of dementia health states was not a logical inconsistency, as for these states, it cannot be determined which is least severe. This is because a given state is not consistently better across all dimensions (e.g., DEMQOL-U states 32143 and 41212). However, this does suggest a difference in the valuation and tradeoff between different dimensions by population, suggesting that a value set estimated for patients and carers would have different relative weightings for different dimensions. Further research is encouraged, as although previous work has suggested that patients and members of the general population weight problems in physical and mental health differently,^16^ there may also be differences in relative weightings between different aspects of mental health and cognition by population. Any difference in the relative weightings of physical and mental health and cognition by members of the general population and patients is important for policy. The use of general population values to inform policy may mean that physical health is given a higher priority than mental health and cognition due to the higher relative weighting of physical health states. This may mean that physical health conditions may be prioritized at the expense of mental health conditions including dementia. This may also mean that mental health and cognition are given a lower priority than patients of these conditions feel is warranted due to their higher relative weightings for mental health over physical health.

Patients and carers had a higher range of mean utility values for dementia health states than the general population. This has important implications for cost-utility analyses, as a larger range is likely to mean a larger change in QALYs over time or across health interventions. In addition, the size of the difference in values between populations for a given dementia health state is large, with coefficients for the interaction terms between population and dementia health states in the regression analysis varying from −0.131 to −0.274 for patients valuing DEMQOL-U and −0.061 to −0.321 for carers valuing DEMQOL-Proxy-U. These differences are large, considering that for the SF-6D, a generic preference-based measure derived from the SF-36, a minimal important difference has been found to range from 0.010 to 0.048 with a mean effect size of 0.051.^26^ Recent trials in dementia, for example, the HTA-SADD trial for the use of antidepressants for depression in dementia,^27^ have included DEMQOL and DEMQOL-Proxy and have generated utility values using DEMQOL and DEMQOL-Proxy-U.^11^ If these values were used to inform resource allocation, the use of general population values would impact the results.

Regression analysis indicated that differences in utility values by population were not due to differences in the observed sociodemographic characteristics of the population. Few sociodemographic variable coefficients were significant and had a minimal impact on model performance and hence were not reported. This indicates that the differences in elicited utility values may be due to a range of other factors such as other unobserved differences in the population, consideration of adaptation, response shift, or differences in their understanding and interpretation of the dementia health states.

The differences in patient and carer values and general population values may reflect the public perception in general of dementia. Dementia is a disorder that attracts substantial stigma and that is relatively poorly understood by the general public including misconceptions that it is a normal part of aging and that there is nothing that can be done to help people with dementia.^28^ This lack of clarity about dementia may drive the higher values provided by the general population for dementia health states compared to the values provided by those affected by dementia who will have a clearer idea of the multiple negative impacts of the disorder. The wording and labeling of the classification system may also have impacted values. Respondents from the general population were not informed that the study was about dementia or that the health states were dementia specific, whereas patients with dementia and carers knew that the study was about dementia and may have realized that the health states were dementia specific. This meant that patients and carers may have had a greater understanding of the dementia health states and also known the underlying cause of the health state. This means that the differences in values across the populations may have been due to the labeling effect or due to the differences in underlying preferences across the different populations, and using our study design, it is not possible to disentangle these 2 effects. This can be interpreted as a weakness of our study design. Indeed, a recent study examining the effects of including a condition label in the description of health states valued using TTO by members of the general population found that condition labels impacted utility values and that the impact differed by condition label and health state severity.^29^ However, we believe that it is an advantage of our study that members of the general population did not know the underlying cause of the health state, as it is clear that there are public misconceptions of cognitive impairment and dementia (e.g., its being an inevitable part of aging) and considerable stigma attached to dementia.^28^ The use of a condition label would mean that these misconceptions and stigma may have inappropriately impacted the utility values elicited from the general population.

One potential limitation of the study is the use of ranking as a warm-up task for members of the general population and no warm-up task for patients or carers. The ranking task was included for members of the general population in accordance with the MVH protocol of TTO used to value the EQ-5D in the UK but was thought too complex for the patient population that has neuropsychological and cognitive problems. The lack of a warm-up task for patients and carers may have affected their values, as prior to the TTO task, they had not had a chance to think about how they would value different dementia health states, and it may have affected their understanding of the TTO task. However, the rank task is a very different task to the TTO task, and the rank task can be seen as a way of encouraging respondents to think about the dementia health states and how they value them in comparison to each other. Arguably, the ranking task was more important for members of the general population who have not experienced these or similar health states and may be unfamiliar with the context of the dementia health state, whereas patients and carers have prior knowledge of similar dementia health states.

Another potential limitation of this study is the requirement of patients with dementia, who by definition have cognitive impairment, to value dementia health states using a cognitively demanding elicitation technique. Patients and carers had significantly higher proportions of respondents (12.7% and 7.0%, respectively) than the general population (1.3% for DEMQOL-U and 0% for DEMQOL-Proxy-U) who were reported by the interviewers as doubtful that they understood the TTO task, but these proportions are not especially high. The patient group was restricted to those with dementia of a mild severity to maximize their ability to complete the elicitation technique. The preferred regression model specifications were also estimated using samples that excluded respondents whose understanding of the TTO task was doubted by the interviewers, but the impact upon the coefficients of dementia health state severity and interaction effects was minimal. The MVH TTO protocol has been widely used in valuation studies of the general population but may be more challenging for respondents with cognitive problems. However, TTO is no more challenging than many other iterative elicitation techniques such as standard gamble or person tradeoff. Ranking and discrete choice-based valuation methods are cognitively demanding in a different way, as the tasks require the simultaneous consideration of multiple health states in which all of the attributes can vary between health states and/or tasks. In addition, ordinal data face the challenge of anchoring the values on the 1-0 full health–dead scale (see Rowen and others^30^ for an overview). Best-worst scaling is one option that may be less cognitively demanding and that has successfully been used in adolescents^31^ but requires additional data, such as TTO or standard gamble data, to anchor the values onto the 1-0 full health–dead scale.

The study was designed to take into consideration the health and competencies of the patient population at every stage: the team engaged with the Alzheimer’s Society through its Quality Research in Dementia panel prior to designing the study; patients were referred to the study by clinicians specializing in dementia; and interviews were immediately terminated if the patient suffered from fatigue, misunderstanding, or distress during the interview. For these reasons, the samples are composed of patients with mild dementia and carers of patients with mild dementia. This means that there may be limits in the generalizability of findings to those with more severe dementia and their carers, as we do not know whether preferences of carers and patients with mild dementia are representative of all carers and patients with dementia. This was a constraint of conducting a valuation study in this patient group.

Patients with dementia and carers of patients with dementia gave systematically lower utility values for dementia health states than members of the general population. The differences in values were not due to differences in the sociodemographic characteristics of the populations. The ordering of utility values for dementia health states differed across populations, suggesting a difference in the valuation and tradeoff between different dimensions across populations. These results suggest that the population used to produce dementia health state utility values could impact the results of cost-utility analyses and potentially affect resource allocation decisions. However, at present, only general population values are available for use to generate utility values for cost-utility analyses. The results of this work suggest that this may lead to higher utility values being used in economic evaluations than patients and carers indicate are representative of dementia health states.
